# Altered DNA Methylation of Long Noncoding RNA uc.167 Inhibits Cell Differentiation in Heart Development

**DOI:** 10.1155/2018/4658024

**Published:** 2018-05-30

**Authors:** Anwen Yin, Mengwen Feng, Zijie Cheng, Qijun Zhang, Hua Li, Jia Xu, Hao Zhang, Yun Li, Lingmei Qian

**Affiliations:** ^1^Department of Cardiology, The First Affiliated Hospital of Nanjing Medical University, Nanjing, China; ^2^Department of Cardiology, Yinzhou Hospital, Medical School of Ningbo University, Ningbo, China; ^3^Department of Pharmacy, Obstetrics and Gynecology Hospital, Nanjing Medical University, Nanjing, China

## Abstract

In previous studies, we have demonstrated the function of uc.167 in the heart development. DNA methylation plays a crucial role in regulating the expression of developmental genes during embryonic development. In this study, the methylomic landscape was investigated in order to identify the DNA methylation alterations. Methylated DNA immunoprecipitation (MeDIP) was performed to examine the differences in methylation status of overexpressed uc.167 in P19 cells. GO and KEGG pathway analyses of differentially methylated genes were also conducted. We found that the distribution of differentially methylated regions (DMRs) peaks in different components of genome was mainly located in intergenic regions and intron. The biological process associated with uc.167 was focal adhesion and Rap1 signaling pathway. MEF2C was significantly decreased in uc.167 overexpressed group, suggesting that uc.167 may influence the P19 differentiation through MEF2C reduction. Taken together, our findings revealed that the effect of uc.167 on P19 differentiation may be attributed to the altered methylation of specific genes.

## 1. Introduction

Congenital heart disease (CHD), one of the most common congenital malformations worldwide, is caused by the dysplasia of heart and vessels in fetal period. The most prevalent CHD subtype is the ventricular septal defects (VSD), making up about 40% of CHD [1]. These are related to a combination of environmental and genetic factors such as NKX2.5, GATA4, TBX5, and MEF2C, which are simply caused by environmental factors only accounting for only 2–5% [2]. Mutations of different genes can lead to different subtypes of CHD, which puts heavy economic and social burdens on the family and society [3].

Epigenetics is regulated by various mechanisms, including histone modifications, DNA methylation, ATP-dependent chromatin-remodeling complexes, and small RNAs, like siRNA, miRNAs, and other noncoding RNAs [4]. DNA methylation is an epigenetic modification associated with transcriptional changes [5]. As an underlying mechanism for birth defects, including CHD, derangements in epigenetic regulated by DNA methylation, can lead to disturbed embryogenesis [6].

Ultraconserved elements (UCEs) are a specific type of long noncoding RNAs and are absolutely conserved between orthologous regions of the human, rat, and mouse genomes [7]. LncRNAs have been shown to influence gene expression by being recruited to gene promoters and modifying the gene imprinting, which is associated with DNA methylation. It has been revealed that changes in DNA methylation may partly disrupt the control of specific promoter usage in cancers [8]. However, the DNA methylation dynamic pattern of long noncoding RNA has remained unknown in heart development.

In previous studies, we have demonstrated the impact of lncRNA uc.167 on the development of embryonic heart and cardiomyocytes' growth, proliferation, apoptosis, and differentiation [9]. Analyzing the methylation changes in cardiomyocytes may provide an insight into the mechanism of uc.167 regulation in CHD. In this study, methylated DNA immunoprecipitation sequencing (MeDIP-seq) was performed to provide extensive coverage of the methylomic landscape in order to identify the different distribution levels of genome-wide methylation regions. In addition, after overexpressing uc.167 in P19 cells, the related biological functions and pathways are discussed. Our research provided evidence for the mechanism underlying uc.167 in the heart development.

## 2. Materials and Methods

### 2.1. Cell Culture and Plasmid Transfection

P19 cells were obtained from the American Type Culture Collection (ATCC, Manassas, VA, USA). The cells were cultivated in Alpha Minimum Essential Medium (*α*-MEM; Gibco, Carlsbad, CA, USA) containing 10% fetal bovine serum (FBS; Gibco), 100 U/ml penicillin, and 50 ug/ml streptomycin at 37°C in 5% CO2. Cells were seeded into 6-well culture plates and grown to 50–70% confluence before transfection, which was performed according to the Lipofectamine 2000 DNA transfection reagent protocol (Life Technologies, Inc., Waltham, MA, USA). The plasmid for overexpression (pCDNA3.1(+)) was constructed by GenePharma (Shanghai, China). The same empty vector plasmid was used as control group.

### 2.2. Real-Time PCR

Primers were designed in National Center for Biotechnology Information (NCBI) and synthesized by Generay (Shanghai, China) (Table S1). The RNA extraction (Tiangen), reverse transcription (Takara 047A), and qRT-PCR (SYBR Green PCR Kit (RR420A)) were performed according to the manufacturer's guidelines. The expressions of uc.167 were quantified to confirm successful overexpression. The data were analyzed using the 2^−ΔΔCT^ method.

### 2.3. Nuclear and Cytoplasmic Fraction

Nuclear and cytoplasmic fractions were divided using a PARIS Kit (Thermo Fisher Scientific). At least 10^7^ cultured cells in total were collected, and then 500 ul ice-cold cell fractionation buffer (CFB) was added. The pellet was gently resuspended by pipetting and continuously incubated on ice for 10 min. The nuclear pellet fraction was separated from cytoplasmic fraction after samples were centrifuged at 500*g* for 5 min. The pellet was washed twice in ice-cold CFB to prevent the structure of cytoplasmic fraction from contaminating the nuclear fraction.

### 2.4. DNA Extraction and Library Preparation

DNA from transfected P19 cells was extracted using TissueLyser and AllPrep DNA/RNA/Protein Mini Kit (Qiagen) according to the manufacturer's protocols. Samples were quantified through 1% agarose gel electrophoresis with Omega Tissue DNA Kit (200) (D3396-02).

### 2.5. Methylated DNA Immunoprecipitation Sequencing Procedure

Covaris S2 system was used to sonicate genomic DNA (3 *μ*g) for 55 s at intensity of 4 for 200 cycles per burst. Illumina library preparation was conducted by using the Sample Preparation Kit. Fragmented DNA was end-repaired and ATP-tailed and the adaptor was ligated. Then the adaptor-ligated DNA was recovered by AMPure XP Beads (Beckman Coulter), denatured, and then subjected to methylated DNA immunoprecipitation (MeDIP). 5 g of a monoclonal antibody against 5-methylcytosine (Eurogentec) coupled to magnetic Dynabeads with M-280 sheep antibody against mouse IgG (Thermo Fisher Scientific) was used to carry out MeDIP. Sequencing libraries were denatured at 95°C for 10 min and incubation with the beads in the IP Buffer (140 mM NaCl, 0.25% Triton ×100, and 10 Mm sodium phosphate buffer (pH 7.0)) was performed at 4°C for 4 h. The IP buffer was used to wash the beads for three times and DNA was eluted in the elution buffer (10 mM EDTA, 1% SDS, and 50 mM Tris-HCl (pH 7.5)) for 15 min at 65°C. Then the beads were conducted with proteinase K at 55°C for 2 h, and methylated DNA was recovered with the QIA quick PCR Purification Kit (Qiagen). Quantitative PCR (qPCR) was used to assess the efficiency of MeDIP. After MeDIP enrichment, remaining DNA was amplified with sequencing primers through PCR and sequenced with the HiSeq 2500 platform (Illumina).

### 2.6. Data Preprocessed and Quality Control

Raw reads including overall the low-quality reads, more than 20 bases and proportion less than 50%, less than 20 bases in 3′ end, joint sequence, reads less than 20 in length, and reads with a nitrogenous preprocessed base were removed (FASTX-Toolkit) (version: 0.0.13). Then we used Bowtie (version: 0.12.8) to conduct genome mapping; the reads were aligned to the reference genomes and then the efficiency of enrichment was calculated. The relationships between the coverage and different depth in the area of CpG island, promoter, and the whole genome were analyzed. The saturation and the efficiency of the methylated enrichment should satisfy the quality control.

### 2.7. Methylation Analysis

MeDIP-Seq peaks were counted using MACS (version: 1.4.2). Control (vector) versus uc.167 peaks were recognized as hypomethylation peaks, and uc.167 versus control (vector) peaks were recognized as hypermethylation peaks. The distribution of peaks in the CpG island, different gene functional components, differences between groups based on RPKM (reads per kilobase per million mapped reads), and the correlations of enrichment between samples were analyzed.

### 2.8. Bisulfate Sequencing PCR (BSP)

Genomic DNA was first treated by bisulfate according to the DNA Bisulfate Conversion Kit protocol (TIANGEN). BSP was performed using methylation-specific primers and PCR Kit (Tiangen). PCR amplifications were performed in 30 uL reaction mixtures containing 2 uL of bisulfite-treated genomic DNA, and the reaction conditions were 5 min at 95°C, 10 cycles for 30 s at 94°C, 30 s at 60°C, and 30 s at 72°C, 25 cycles for 30 s at 94°C, 30 s at 50°C and 30 s at 72°C, and finally at 60°C for 30 min. The PCR products were separated on 2% agarose gels and cloned into a pCRII vector using TA Cloning Kit, Dual Promoter (Invitrogen). Plasmids DNA from 10 colonies were sequenced. Primers were listed in Table S1.

### 2.9. Gene Ontology (GO) Analysis

Data were analyzed based on GO terms. The GO stats package was applied to test overrepresented GO terms [10]. The hypergeometric test was applied to filter the significant enrichment of differentially methylated region (DMR) [11]. The calculated *P* value is corrected by Bonferroni test, and the GO term that satisfies the condition that corrected *P* value (FDR) ≤ 0.05 (FDR = 1 − Nk/T) [12] is defined as the GO term that is significantly enriched in the expression of the differential genes (Nk: the number of Fisher's test *P* values less than v2 test *P* values). GO analysis was used to analyze the main function of the differential expression genes according to the GO database that offers the key functional classifications to NCBI.

### 2.10. Pathway Analysis

Pathway analysis is a functional analysis mapping genes to KEGG pathways. We select the significant pathway based on the *P* value taht denotes the significance of the pathway correlated to the conditions [12]. The lower the *P* value is, the more important the pathway is. This approach also identifies functional relationships among genes, such as upregulation, downregulation, or direct binding.

### 2.11. Statistical Analysis

Using SPSS 13.0 software, all the values are presented as the means ± standard error of the mean (SEM). Statistical analyses were conducted with Student's *t*-test. The statistical significance was *P* < 0.05.

## 3. Results

### 3.1. The Establishment of Stable P19 Cells Lines Overexpressed uc.167

**Figure 1:**
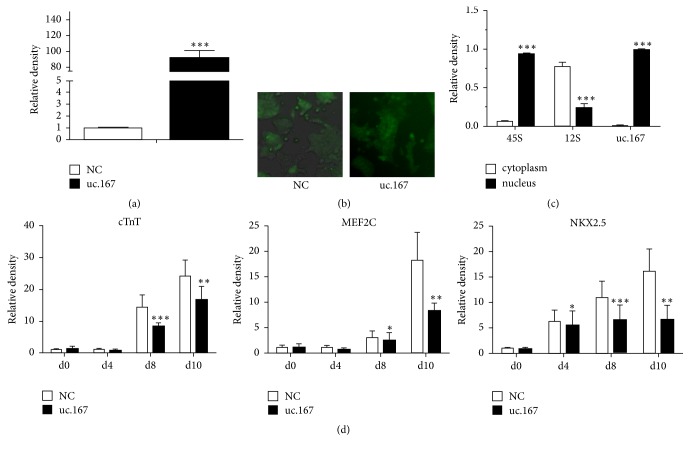
Locations and functions of uc.167. (a) uc.167 expression levels in P19 cells were significantly higher than those of the NC groups. ^*∗∗∗*^*P* < 0.001 and ^*∗∗*^*P* < 0.01, Student's *t*-test. (b) Optical microscopy and fluorescence microscopy (×100) were used to observe lentivirus transfection efficiency via GFP expression of the uc.167 overexpression vector and control vector in P19 cells after 48 h transfection. (c) qRT-PCR analyses of uc.167 in cytoplasmic and nuclear fractions from P19 cells. uc.167 mainly located in the nucleus. (d) Cardiac markers were decreased in the uc.167 overexpressed group compared with control group. ^*∗*^*P* < 0.05 versus NC groups. Student's *t*-test.

To explore the methylation effects of uc.167, we constructed uc.167 overexpression plasmids. The transfection efficiency was detected through quantitative analysis that demonstrated that there was approximately 80-fold excess in P19 cells overexpressing uc.167 compared to control group (Figures 1(a) and 1(b)).

### 3.2. Location of uc.167

Nuclear and cytoplasmic fractionation of P19 cells manifested that uc.167 was mainly localized in the nucleus compartment (Figure 1(c)). As a control, the 45S rRNA precursor was primarily localized to the nuclear fraction, whereas the mitochondrial 12S rRNA gene was mainly found in the cytoplasmic compartment. Overexpressed uc.167 influenced P19 cells differentiation to cardiomyocytes* and caused a significant decrease of cardiac markers including cTnT (d8,d10), MEF2C (d8, d10), and NKX2.5 (d4, d8, d10) (Figure 1(d)).*

### 3.3. Characterization of Differential Methylated Regions

**Figure 2:**
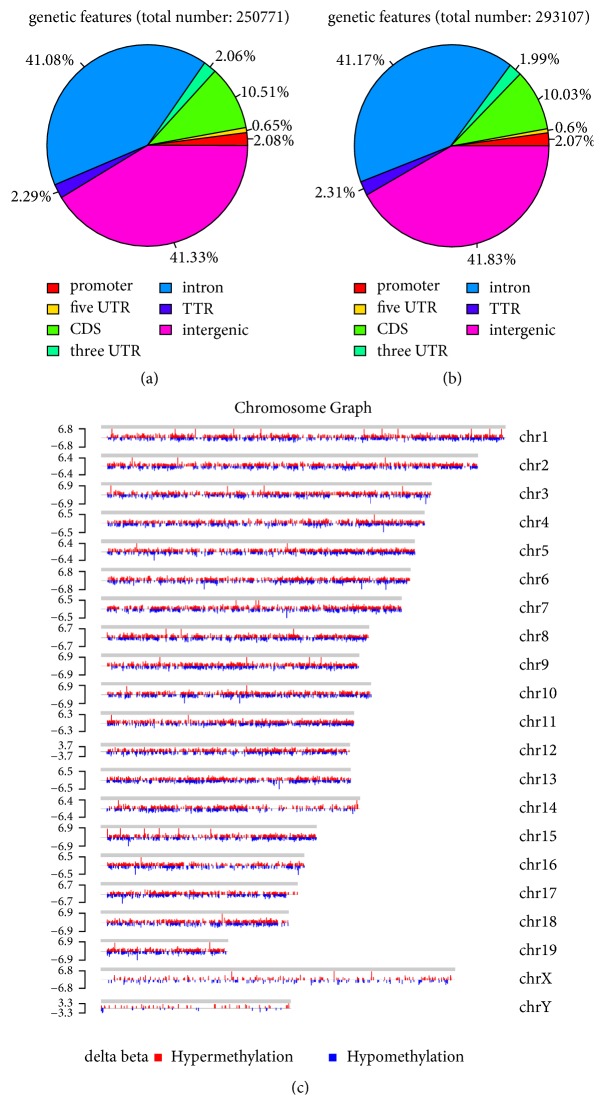
Differential methylated regions and chromosomes. (a) DMRs peaks distribution in NC group. (b) DMRs peaks distribution in uc.167 overexpressed group. (c) Hypomethylated and hypermethylated peaks distribution in chromosomes.

To identify the differentially methylated genes in uc.167 overexpression cells, we initially classified the CpGs according to their methylation status. The DMRs were located in promoter, 3′UTR, 5′UTR, CDS, intron, TTR, and intergenic regions, respectively. The DMRs peaks distributed in different components of genome showed that reads in intergenic regions (41.33% in NC group and 41.83% in overexpressed uc.167 group) and intron (41.08% in NC group and 41.17% in overexpressed uc.167 group) had a relatively higher methylation level than others (Figures 2(a) and 2(b)). The distribution of differentially methylated genes in chromosome was also shown in Figure 2(c).

### 3.4. GO Analysis

**Figure 3:**
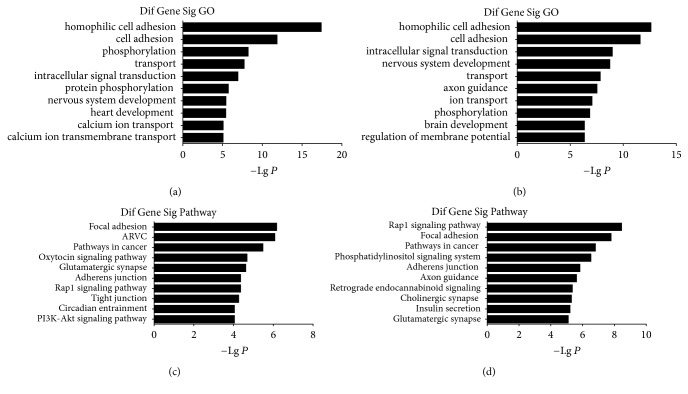
GO and KEGG analysis for the differentially methylated genes in uc.167 overexpressed groups. (a) GO analysis of hypermethylated genes. (b) GO analysis of hypomethylated genes. (c) KEGG pathways analysis of hypermethylated genes. (d) KEGG pathways analysis of hypomethylated genes.

The ontologies and gene associations can be accessible from the GO website (http://www.geneontology.org). Interestingly, we observed that the genes that were hypermethylated in uc.167 overexpression P19 cells were potentially relevant to (1) homophilic cell adhesion via plasma membrane adhesion molecules, (2) cell adhesion, (3) phosphorylation, (4) transport, (5) intracellular signal transduction, (6) protein phosphorylation, (7) nervous system development, (8) heart development, (9) calcium ion transport, and (10) calcium ion transmembrane transport (Figure 3(a)). Meanwhile, the enriched GO terms associated with the hypomethylated genes in uc.167 overexpression P19 cells were involved in (1) homophilic cell adhesion via plasma membrane adhesion molecules, (2) cell adhesion, (3) intracellular signal transduction, (4) nervous system development, (5) transport, (6) axon guidance, (7) ion transport, (8) phosphorylation, (9) brain development, and (10) regulation of membrane potential (Figure 3(b)). Overall, these results suggest that these biological processes are epigenetically regulated in uc.167 overexpression P19 cells.

### 3.5. Pathway Analysis

**Figure 4:**
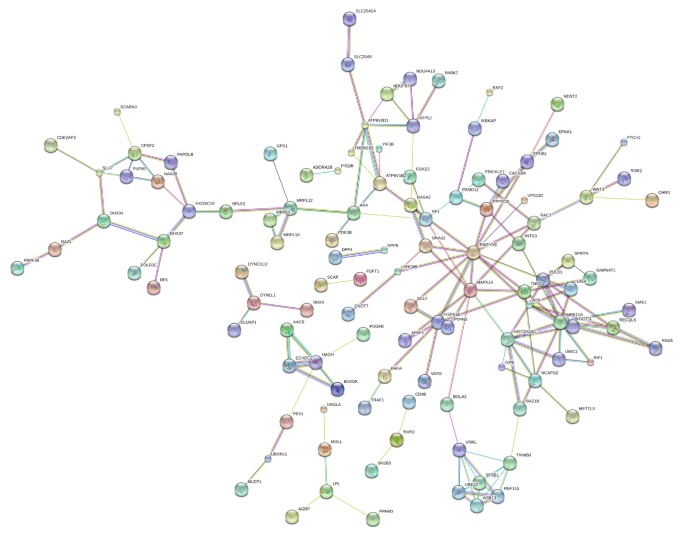
Interactions between the differentially methylated genes in uc.167 overexpressed group and control group, as identified using the biological network analysis function of Ingenuity Pathway Analysis.

To further characterize the functional significance of the differentially methylated genes, we performed a systematic analysis and searched pathways that were significantly enriched in the uc.167 overexpressed groups. The pathway diagrams are used to catch how genes regulate and interact with each other. KEGG pathway analysis showed that hypermethylated genes were implicated in the following pathways: (1) focal adhesion, (2) arrhythmogenic right ventricular cardiomyopathy (ARVC), (3) pathways in cancer, (4) oxytocin signaling pathway, (5) glutamatergic synapse, (6) adherens junction, (7) Rap1 signaling pathway, (8) tight junction, (9) circadian entrainment, and (10) PI3K-Akt signaling pathway (Figure 3(c)). KEGG pathway analysis also showed that hypomethylated genes were mainly involved the following pathways: (1) Rap1 signaling pathway, (2) focal adhesion, (3) pathways in cancer, (4) phosphatidylinositol signaling system, (5) adherens junction, (6) axon guidance, (7) retrograde endocannabinoid signaling, (8) cholinergic synapse, (9) insulin secretion, and (10) glutamatergic synapse (Figure 3(d)). Based on this computed signaling network, we found that multiple signaling procedures, including Rap1, PI3K-Akt, and phosphatidylinositol signaling system, were important to the formation of this pathway network (Figure 4).

### 3.6. Analysis of DNA Methylation in P19 Cells

**Figure 5:**
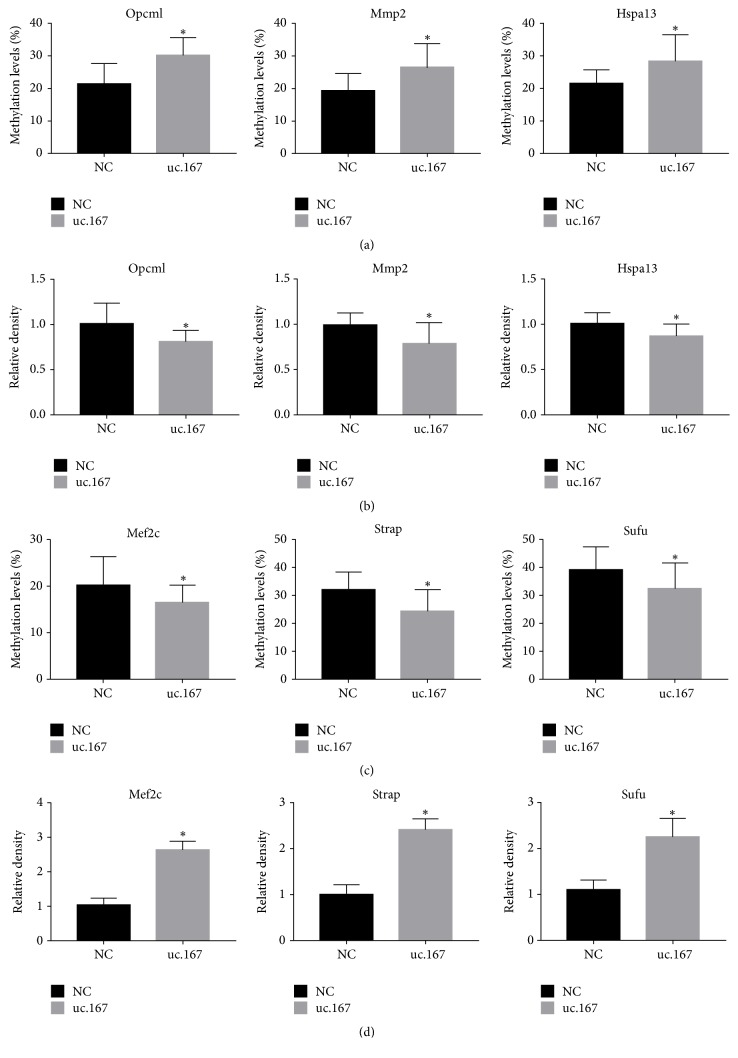
The levels of promoter methylation were measured by BSP in uc.167 overexpressed and control cells. (a) The methylation level was increased in Opcm1, Mmp2, and Hspa13. (b) The mRNA level was decreased in Opcm1, Mmp2, and Hspa13. (c) The methylation level was decreased in MEF2C, Strap, and Sufu. (d) The mRNA level was increased in MEF2C, Strap, and Sufu.

To confirm that the microarray data correctly reflected the differences in methylation, we examined the levels of promoter methylation for several genes. We quantitatively measured site-specific CpG methylation upstream of Opcm1, Mmp2, Hspa13, MEF2C, Strap, and Sufu. Microarray analysis predicted the promoter regions of Opcm1, Mmp2, and Hspa13 to be hypermethylated in uc.167 overexpression groups, while the promoter regions of MEF2C, Strap, and Sufu were predicted to be hypomethylated. BSP confirmed that the site-specific CpGs of Opcm1, Mmp2, and Hspa13 were hypermethylated (Figure 5(a)) and the relative mRNA levels were significantly decreased in the uc.167 overexpression group (Figure 5(b)). We also verified the site-specific CpGs of MEF2C, Strap, and Sufu which were hypomethylated (Figure 5(c)) and the relative mRNAs level increased in uc.167 overexpression groups (Figure 5(d)), although the absolute differences were generally small.

## 4. Discussion

The molecular network of embryonic heart development, however, is still unclear. In previous research, we have demonstrated that overexpression of uc.167 promoted apoptosis and inhibited proliferation in embryonic myocardial cells [9]. In this study, we analyzed the DNA methylation alterations in uc.167 overexpressed P19 cells and revealed methylation alterations in specific genes participating in the mechanism underlying the function of uc.167 in heart development.

Three main epigenetic processes are represented by DNA methylation, posttranslational histone modifications, and RNA-based mechanisms [13]. Dysregulated epigenetic modifications may lead to irregular development of disease. Indeed, DNA methylation has been associated with insulin sensitivity [14], coronary heart disease [15, 16], obesity [17], and breast cancer [18]. DNA methylation is usually associated with transcriptional suppression, while demethylation is associated with transcriptional activation, thus affecting gene expression by modifying DNA promoter approachability to RNA polymerase and gene level. The decrease in DNA methylation in the promoter region of lncRNA H19 also was associated with calcific aortic valve disease (CAVD) [19]. Such changes were shown to promote an osteogenic program by interfering with the expression of NOTCH1. The altered promoter methylation in genes relevant to myocyte apoptosis, fibrosis, and contractility was revealed in patients with heart failure [20]. The present findings shed new light on the first time that lncRNA, uc.167, is dysregulated and contributes to the aberrant heart development. LncRNA uc.167 could represent a novel target in congenital heart disease.

During embryo development, the heart is one of the most significant organs, and a series of complex morphogenetic interactions contribute to the development of the heart [21]. A balance between cardiomyocyte apoptosis and proliferation determines the growth of the embryonic fetal heart [22]. In mammalian cells, DNA methylation is catalyzed by DNMTs; DNA methylation has a crucial impact on the regulation of gene expression and maintaining genomic integrity [23]. LncRNAs are known to regulate the expression of adjacent genes as* cis*-acting regulatory elements [24]. In the previous study, expression of uc.167 showed an opposite correlation with MEF2C in the process of P19 cells differentiation [9]. Overexpression of MEF2C partially reverses the negative effects of uc.167 on proliferation, apoptosis, and differentiation. In this study, the methylation level of MEF2C was decreased compared with control groups, suggesting that uc.167 may influence the P19 differentiation through altering MEF2C methylation status.

The genome-wide epigenetic studies will allow identification of unique regulatory pathways, giving new insight into the etiology of congenital heart disease such as ventricular septal defects.

## Supplementary Materials

Supplementary Materials Table S1: primers used in this study.
