# Supplementary material for: Altered DNA Methylation of Long Noncoding RNA uc.167 Inhibits Cell Differentiation in Heart Development
Source: Biomed Res Int. 2018 May 30;2018:4658024. doi: 10.1155/2018/4658024 (PMC5998154; doi:10.1155/2018/4658024)
Supplement: Supplementary Materials — Table S1: primers used in this study. [file 4658024.f1.docx]

**Table S1. Primers used in this study.**

| **Primer** | **Sequence** | **Application** |
| --- | --- | --- |
| uc.167 | F: TCACCCCTCCAGTCTCAACTA  R: AGCAAAATGTCAGGAATGGAAAGA | qPCR |
| Mef2c | F: CCAGCACTGACATGGATAAGG  R: CTGCCAGGTGGGATAAGAACG | qPCR |
| cTnT | F: TGAGACAGAGGAGGCCAACGTAG  R: CTGCCTTTCCTTCTCCCGCTCAT | qPCR |
| 45S | F: GTGCCCTCACGTGTTTCACTTT  R: TAGGAGACAAACCTGGAACGCT | qPCR |
| 12S | F: AAGGTTTGGTCCTGGCCTTA  R: GTGGCTAGGCAAGGTGTCTT | qPCR |
| Strap | F: GCATCACGCCTTACGGCTA  R: AATCCAGTCTCCTGTATCTCCC | qPCR |
| Adtb2 | F: GCATCACGCCTTACGGCTA  R: AATCCAGTCTCCTGTATCTCCC | qPCR |
| Nkx2.5 | F: CCTCCGCCAACAGCAACTT  R: TCTGAGGGACAGGGCATAGTG | qPCR |
| Opcml | F: CCCGCCTACTGGATCGTCT  R: CATCTATGGTACACCTGAGGGT | qPCR |
| Mmp2 | F: ACCTGAACACTTTCTATGGCTG  R: CTTCCGCATGGTCTCGATG | qPCR |
| Hspa13 | F: GCCGGAGAGATGACGATCTTA  R: TTGGCGTAGGCAATGGTAAAT | qPCR |
| Opcml | F:AAGGTGGTTAATATATTATAAAATGAATAT  R: AAAAAATTACTTAAACCAACCAAAC | BSP |
| Mmp2 | F: TTTTTGAATGATTATTGGGTATATAA  R: TTAAAATATCAATTTTAAATCTTTCCTACT | BSP |
| Hspa13 | F: TTATAGAGGTAGAATTTGAGGGTTT  R:CACAAAATATTCTAACAAAAAAACTATATC | BSP |
| Mef2c | F: TTGTAATTTTAGTATTTTGGGAGGT  R: ATCCTAATTTATTTATTTATTTATTTATTT | BSP |
| Strap | F: GTTTTAGTTTTGGTTTGTTTTTTTT  R: ATAATAATACCATCCTCAAAATTTTATTTA | BSP |
| Sufu | F: TGATTGATGTTTTGATTTTGTTTTT  R: CCCATAATTCCCCACTCTTAAC | BSP |
